# Predictive risk modelling in the Spanish population: a cross-sectional study

**DOI:** 10.1186/1472-6963-13-269

**Published:** 2013-07-09

**Authors:** Juan F Orueta, Roberto Nuño-Solinis, Maider Mateos, Itziar Vergara, Gonzalo Grandes, Santiago Esnaola

**Affiliations:** 1 Osakidetza, Basque Health Service, Bilbao, Spain; 2 O + berri, Basque Institute for Healthcare Innovation, Plaza Asua 1, 48150, Sondika, Spain; 3 Department of Health and Consumer Affairs, Basque Government, Vitoria, Spain

**Keywords:** Risk-adjustment, Burden of illness, Actuarial prediction, Health risk stratification

## Abstract

**Background:**

An increase in chronic conditions is currently the greatest threat to human health and to the sustainability of health systems. Risk adjustment systems may enable population stratification programmes to be developed and become instrumental in implementing new models of care.

The objectives of this study are to evaluate the capability of ACG-PM, DCG-HCC and CRG-based models to predict healthcare costs and identify patients that will be high consumers and to analyse changes to predictive capacity when socio-economic variables are added.

**Methods:**

This cross-sectional study used data of all Basque Country citizens over 14 years of age (n = 1,964,337) collected in a period of 2 years. Data from the first 12 months (age, sex, area deprivation index, diagnoses, procedures, prescriptions and previous cost) were used to construct the explanatory variables. The ability of models to predict healthcare costs in the following 12 months was assessed using the coefficient of determination and to identify the patients with highest costs by means of receiver operating characteristic (ROC) curve analysis.

**Results:**

The coefficients of determination ranged from 0.18 to 0.21 for diagnosis-based models, 0.17-0.18 for prescription-based and 0.21-0.24 for the combination of both. The observed area under the ROC curve was 0.78-0.86 (identifying patients with a cost higher than P-95) and 0.83-0.90 (P-99). The values of the DCG-HCC models are slightly higher and those of the CRG models are lower, although prescription information could not be used in the latter. On adding previous cost data, differences between the three systems decrease appreciably. Inclusion of the deprivation index led to only marginal improvements in explanatory power.

**Conclusion:**

The case-mix systems developed in the USA can be useful in a publicly financed healthcare system with universal coverage to identify people at risk of high health resource consumption and whose situation is potentially preventable through proactive interventions.

## Background

Increased life expectancy combined with other factors has produced a progressive increase in the prevalence of chronic diseases and multimorbidity situations, especially in the older population strata. However, current healthcare systems were designed to serve primarily acute episodes of illness and have trouble meeting the complex healthcare needs that this group of people present [1]. In addition, a small number of patients with multiple pathologies requires such a high number of recurrent hospitalisations and other costly treatments that the cost of their care accounts for the majority of the budgets of health organisations.

In this context, in 2010 the Basque Government’s Department of Health published a Strategy to tackle the challenge of Chronicity in the Basque Country [2], containing a series of policies and projects to reinvent the healthcare delivery model and adapt it to this new situation. In order for interventions to be effective and efficient, they should be implemented among those patients whose care needs match the profile for which they were designed. This fact raises the need to develop a population stratification system based on risk adjustment mechanisms.

The risk adjustment models use information obtained from patients to explain the variation in healthcare resource consumption, cost and the outcomes of the care they receive. To do this, models were developed using different explanatory variables such as demographics, past consumption of health resources and health status [3-7]. Incorporating clinical variables generates greater explanatory power. Moreover, systems that contain these variables are easier to interpret for the healthcare professionals responsible for caring for these patients.

Among the best-known predictive instruments are Adjusted Clinical Groups (ACG-PM) [8], Diagnostic Cost Groups/Hierarchical Condition Categories (DCG-HCC) [9] and Clinical Risk Groups (CRG) [10]. All three were designed in the U.S., and are robust systems from a statistical point of view and versatile in their applications. Their usefulness has been proven in public and private health organisations over a number of years. They are able to explain a large portion of the variability in a population’s use of health services and to provide a forecast estimate of the volume of healthcare resources that each individual will require the following year. The most recent versions combine information about diagnoses, prescriptions, previous cost and use of certain procedures.

Despite their undeniable appeal, these instruments also have some limitations resulting from their failure to include other factors that influence health, such as lifestyle, socio-economic variables and other factors relating to the social environment [11,12].

There may also be doubts about the validity and applicability of these instruments in our setting. Although several studies have demonstrated the ability of diagnosis-based case-mixes to retrospectively explain the use of health resources in Spain [13,14] and other countries, there are few references comparing different predictive tools in a national system similar to ours. In addition, the limited development of health databases for administrative purposes in Spain may pose obstacles to the implementation of risk prediction models.

This paper has two objectives. First, to verify the validity of ACG-PM, DCG-HCC and CRG systems in terms of predicting healthcare costs and identifying in advance those patients that will consume a high level of resources the following year; and second, to analyse the potential improvement in the predictive capacity of these instruments when variables related to patients’ socio-economic status are incorporated.

## Methods

This is a cross-sectional study carried out within the health system of the Basque Country (Spain). The Spanish National Health System (SNS) provides universal coverage. This coverage and the benefits package are common to Spanish citizens and foreign nationals within Spanish national territory.

The SNS is publicly funded through general taxes. At the point of delivery, provision is free of charge, with the exception of pharmaceuticals prescribed, which entail a co-payment.

The regional organizational structure is the result of a devolution process. Geopolitically, Spain is made up of 17 regions referred to as Autonomous Communities. The 17 regional health ministries have primary control over the funding, organization, and delivery of health services within their territory. These competencies were transferred over the past 30 years and, in particular, the Basque Health Service, called Osakidetza, was created in 1983.

In the Basque Country, there is a purchaser provider split, with the Department of Health and Consumer Affairs of the Basque Government being responsible for policy making, for public health and for planning and financing health care. Osakidetza is the only public provider of health services in the region, including primary care, hospital care (both acute and long-term care), specialist outpatient clinics, emergencies, and mental health. All health professionals in Osakidetza are salaried.

Primary Care is structured in Primary Care Areas, in which primary care is provided through one or more Health Centers under criteria designed to achieve a balance between optimum accessibility and managerial efficiency. There are a total of 1835 doctors working in primary care (1544 general practitioners [GPs] and 291 pediatricians); they work in teams and act as gatekeepers for the other levels of care. Each citizen is on the list of a given primary care doctor and nurse [15].

The study population was composed of all persons over 14 years of age, registered in Osakidetza on 1 September 2008. The study period corresponds to two consecutive 12-month intervals. Data from the first year (01 September 2007 to 31 August 2008) were used to develop the explanatory variables and those from the second year (01 September 2008 to 31 August 2009) for the response variables. A minimum monitoring period was established in the first year, including only those people that were assigned to a doctor in Osakidetza for at least 6 months, regardless of whether or not they had any contact with the health services (n =1,973,971). Of these, 28,182 people did not complete the second follow-up year due to death (n = 18,548), transfer or other causes (n = 9,634). Those citizens in the study population who died during the second year were included, whereas those who withdrew for other reasons were not. As such, the final population consisted of 1,964,337.

### Sources of information

Case-mix systems are used in the USA to classify the population from claim data. Since this information system is not operative in Spain, data were extracted from the available sources. For this study, we obtained permission from the Basque Health Service to use the Basque Country population stratification program (PREST) database. PREST uses an opaque identifier to ensure patient confidentiality and contains information from primary care electronic medical records (PC-EMR), minimum basic data set of hospital discharge reports (MBDS) and computerised files from day hospitals, emergency departments and specialised outpatient offices. These data were as follows:

– Demographic data: age, sex and census area of residence

– ICD-9-CM codes for diagnoses from each contact with primary care, hospital admissions and day hospitals. The latter two also contained information on procedures. The coding is done in hospitals by clinical documentation specialists, but in primary care the doctors themselves must perform this task when establishing or modifying a diagnosis. A more detailed description of this process can be found elsewhere [16]. In order to avoid the possible inclusion of long-term diseases that were not currently active, we only included the diseases that were considered reason for encounter, according to the annotations of the physicians, and involved in some clinical action, such as cause of prescription, new clinical notation or derived visit, during the period of study.

– Prescriptions in primary care, which are coded automatically in the PC-EMRs according to the World Health Organization (WHO) Anatomical Therapeutic Chemical (ATC) system [17].

Regarding the cost, this was obtained directly for primary care prescriptions recorded in PC-EMR. In the other cases (visits to emergency departments, outpatient specialty care and primary care doctors and nurses; lab tests and X-rays requested in primary care; some procedures such as dialysis, radiation therapy or chemotherapy performed in day hospitals) the number of services provided to each patient was multiplied by their standard cost (the average cost of each service provided to a patient treated in Osakidetza, according to calculations made by the aforementioned organisation). The costs of hospital stays and major outpatient surgical procedures were calculated according to the weights of their corresponding Diagnosis Related Groups (DRGs). Services for which no information was available were excluded from the cost estimate: Mental Health (both admissions and outpatient visits), hospital-at-home services and day hospitals (except the above-mentioned procedures), outpatient rehabilitation, medical transport, prostheses and other equipment delivered to patients at home. It was estimated that the total cost of excluded services corresponded to 28.2% of the overall budget.

The deprivation index of the census tract (median population size = 1,200 inhabitants) of residence proposed by the MEDEA project [18] was used as a proxy of individual socioeconomic position. Five simple indicators were included in this index (year 2001): Unemployment, low educational level, low educational level in young people (16–29 years), manual workers, and temporary workers. The deprivation index was categorised in quintiles.

### Classification systems for patients

ACG-PM version 9.0, CRG version 1.6 and DCG-HCC (models ID#26, ID#69 and ID#71) were used for this study. A brief description of them can be found as Additional file 1:

– CRGs [10] is a cell-based model in which each person is assigned to one mutually exclusive category, based on clinical criteria; the total number of CRGs is 1,076. In this study it was not possible to add information on prescriptions, since the Basque country organisation uses the WHO ATC system, which is rejected by the CRG v1.6 software.

– DCG-HCC [9] are a regression-model. All diagnoses and prescriptions are classified into clinically homogeneous groups that are employed to predict the cost of each patient. In this study, 117 categories for diagnoses and 203 for prescriptions were included as independent variables.

– ACG-PM [8] adopts a mixed approach. Patients are categorised in mutually exclusive categories, of which the model uses 34. Other markers are incorporated alongside them, with the complete model comprising 180 variables generated from diagnoses and 65 from prescriptions.

### Statistical models

From the information obtained with the use of the three case-mixes, several regression models were constructed using the cost of the second year as the outcome variable. To avoid overfitting problems and to confirm that the results do not depend on the sample, a fivefold cross validation was carried out. Thus, the sample was split into five random subsamples and model fitting was performed five times, considering four of these subsamples as the training set, and the remaining one as the test set, each time. The statistics employed to evaluate and compare the performance of the different models were derived from this cross validation.

The coefficient of determination R2 was used to measure the explanatory power. First, adjustments were made to models whose independent variables were the out-of-the-box risk scores offered directly by the case-mix developers. Then multiple regression models were recalibrated to our population dataset, in which clinical variables (groups based on diagnoses or drugs from the case-mix), previous cost and socio-economic variables (deprivation index) were successively added to the demographic variables (age and sex).

OLS (Ordinary Least Squares) statistical models do not adequately match the analysed data, as none of the variables follows a normal distribution and additionally, they do not take into account the hierarchical nature of the data (patients grouped in lists of primary care doctors, those working in health centres and centres clustered in health districts). Therefore, two-part models and hierarchical models were also used, including the 3 levels of grouping listed above. Because these models do not directly provide an R2, their results were compared with those obtained by OLS using MAPE (Mean Absolute Prediction Error), expressed as a percentage (dividing the obtained value by the observed average cost).

In order to check the validity of these systems to identify people that will require high resource consumption the following year, logistic regression models were developed in which the dependent variables indicate whether the person belongs to 5% and 1% of highest-consuming patients during the second period. The explanatory variables were the same as in the linear models described above and the area under the ROC curve was the measure used to evaluate and compare the different models.

## Results

Of the study population, 51.2% are women and 21.5% are aged over 65 years. The distribution of the population in age and sex groups, and the averages for unique prescriptions and diagnoses per patient can be found in Table 1. More than three quarters of the population had at least one contact with health services and over 7% required one or more hospitalisations per year. The overall average annual consultations per patient ranged between 0.31-0.32 for emergencies, 1.70-1.76 for outpatient specialty care, 4.29-4.47 for primary care doctors and 1.69-2.09 for nursing consultations. A table in Additional file 2 the distribution of these averages by age group.

**Table 1:** Distribution of the population in age and sex groups and descriptive information on diagnoses and prescriptions

	**Men**	**Women**	**Mean unique diagnoses per patient**	**% Patients with one or more diagnosis**	**Mean drugs prescribed (unique ATC codes) per patient**	**Patients with one or more drugs prescribed**
**Age groups**	**N (%)**	**N (%)**				
14-24	104,686 (5.33%)	99,969 (5.09%)	1.58	52.06%	1.98	65.69%
25-34	178,767 (9.10%)	171,317 (8.72%)	1.73	51.97%	2.12	64.46%
35-44	194,187 (9.89%)	187,426 (9.54%)	1.93	52.77%	2.20	64.04%
45-54	164,620 (8.38%)	165,997 (8.45%)	2.55	58.28%	2.56	67.01%
55-64	135,889 (6.92%)	138,760 (7.06%)	4.29	73.33%	3.54	77.06%
65-74	95,032 (4.84%)	107,207 (5.46%)	6.54	84.62%	4.63	84.06%
75-84	67,272 (3.42%)	95,343 (4.85%)	7.88	86.48%	5.45	85.34%
85+	17,091 (0.87%)	40,774 (2.08%)	6.54	72.44%	4.60	72.38%
Total	957,544 (48.75%)	1,006,793 (51.25%)	3.00	63.01%	3.40	70.68%

Table 2 shows the results obtained by different linear regression models in terms of R [2]. While the age and sex-based models explained 7% of the cost variability, the out-of-the-box models reached values between 13.5% and 20.8%. Regarding those that were recalibrated with our data, the coefficients of determination ranged from 0.18 to 0.21 for diagnosis-based models, 0.17-0.18 for prescription-based and 0.21-0.24 for the combination of both. In all cases the values of the DCG-HCC models are slightly higher and those of the CRG models lower, although prescription information could not be used in the latter. When data on the cost of the previous year is added, differences between the three systems decreased appreciably.

**Table 2:** Coefficients of determination (R2) for the case-mix based models to predict the health cost per patient

		**Demographic variables only**	**ACG-PM**	**CRG**	**DCG-HCC**
Out of the box	Variables:				
	A&S + dx + rx + cost percentiles (age < 65)		0,167		
	A&S + dx + rx + cost percentiles (age 65+)		0,164		
	A&S + dx			0,135	
	A&S + dx + cost				0,181
	A&S + dx + rx + cost				0,208
Recalibration	Variables				
	Age and Sex (A&S):	0,071			
	A&S + Only Dx		0,182	0,181	0,213
	A&S + Only Rx		0,165	-	0,178
	A&S + Dx + Rx		0,211	-	0,236
	A&S + Dx + Rx + cost percentile		0,231	0.218*	0,246
	A&S + Dx + Rx + cost		0,260	0.254*	0,269
	A&S + Dx + Rx + cost + DI		0,260	0.254*	0,269

*The CRG models do not include prescriptions.

Age groups: 14–17; 18–34; 35–44; 45–54; 55–64; 65–69; 70–74; 75–79; 80–84; 85 + .

Cost groups in percentiles: 0–1; 2–10; 11–25; 26–50; 51–75; 76–90; 91–93; 94–95; 96–97; 98 + .

*Dx* diagnoses, *Rx* prescriptions, *DI* Deprivation index.

Table 3 compares the MAPE of the OLS, two-part and hierarchical models. The two-part model shows the worst results and the differences between the OLS and hierarchical models are small and in both directions.

**Table 3:** MAPE (Mean absolute prediction error) expressed as a percentage of the average cost of different models based on case-mix systems, using different regression models

		**Linear regression (OLS)**			**Two-part**			**Hierarchical models**		
		**ACG-PM**	**CRG**	**DCG-HCC**	**ACG-PM**	**CRG**	**DCG-HCC**	**ACG-PM**	**CRG**	**DCG-HCC**
Recalibration	Variables									
	Age and Sex (A&S)		106.80%			109.09%			106.52%	
	A&S + Only Dx	92.36%	96.36%	91.26%	114.59%	104.74%	109.74%	95.27%	95.97%	91.52%
	A&S + Only Rx	93.57%		91.87%	113.84%		115.74%	93.74%		91.98%
	A&S + Dx + Rx	88.41%		87.02%	114.96%		114.44%	89.87%		87.26%
	A&S + Dx + Rx + cost percentile	85.99%	87.70%	85.05%	98.52%	96.11%	97.92%	86.97%	87.72%	85.37%
	A&S + Dx + Rx + cost percentile + DI	86.02%	87.70%	85.06%	98.49%	96.09%	97.89%	86.97%	87.72%	85.37%

*Dx* diagnoses, *Rx* prescriptions, *DI* Deprivation index.

With regard to the models’ capability to correctly identify patients located above the 95th and 99th percentiles, the results are presented in Tables 4 and 5. The out-of-the-box models for the three case mixes produce different AUC values: those for the CRG models are similar to those that use only age and sex and the same applies to the ACG model for patients over 65; the values from the ACG model for younger patients are higher than both of the above, but the highest of all are derived from the DCG-HCC models. In the case of the recalibrated models, the AUC values for the DCG-HCC and ACG-based models display similar behaviour and the AUC values from CRG models are lower.

**Table 4:** Area under the receiver operating characteristics (ROC) curve of the case-mix based logistic regression models to identify patients located above the 95th percentile of health spending

	**Demographic variables only**		**ACG-PM**		**CRG**		**DCG-HCC**	
**Out of the box**	**AUC**	**95% CI**	**AUC**	**95% CI**	**AUC**	**95% CI**	**AUC**	**95% CI**
Variables:								
A&S + dx + rx + cost percentiles (age < 65)			0,816	(0.813 - 0.818)				
A&S + dx + rx + cost percentiles (age 65+)			0,761	(0.760 - 0.764)				
A&S + dx					0,787	(0.786 - 0.789)	0,824	(0.822 - 0.825)
A&S + dx + rx + cost							0,852	(0.851 - 0.853)
Recalibration								
Variables:								
A&S (Age and sex)	0,774	(0.773 - 0.776)						
A&S + Only Dx			0,845	(0.844 - 0.847)	0,797	(0.795 - 0.798)	0,846	(0.845 - 0.848)
A&S + Only Rx			0,833	(0.831 - 0.834)			0,837	(0.836 - 0.838)
A&S + Dx + Rx			0,847	(0.853 - 0.856)			0,858	(0.857 - 0.859)
A&S + Dx + Rx + cost percentile			0,868	(0.866- 0.869)	0.848*	(0.847 - 0.849)	0,868	(0.867 - 0.869)
A&S + Dx + Rx + cost percentile + DI			0,868	(0.867 - 0.869)	0.848*	(0.847 - 0.850)	0,868	

*The CRG models do not include prescriptions.

Age groups: 14–17; 18–34; 35–44; 45–54; 55–64; 65–69; 70–74; 75–79; 80–84; 85 + .

Cost groups in percentiles: 0–1; 2–10; 11–25; 26–50; 51–75; 76–90; 91–93; 94–95; 96–97; 98 + .

*Dx* diagnoses, *Rx* Prescriptions, *DI* Deprivation index.

**Table 5:** Area under the receiver operating characteristics (ROC) curve of the case-mix based logistic regression models to identify patients located above the 99th percentile of health spending

	**Demographic variables only**		**ACG-PM**		**CRG**		**DCG-HCC**	
**Out of the box**	**AUC**	**95% CI**	**AUC**	**95% CI**	**AUC**	**95% CI**	**AUC**	**95% CI**
Variables:								
A&S + dx + rx + cost percentiles (age < 65)			0,852	(0.847 - 0.857)				
A&S + dx + rx + cost percentiles (age 65+)			0,779	(0.774 - 0.783)				
A&S + dx					0,814	(0.811 - 0.818)	0,858	(0.8556 - 0.861)
A&S + dx + rx + cost							0,882	(0.880 - 0.885)
Recalibration								
Variables:								
A&S (Age and sex)	0,811	(0.809 - 0.814)						
A&S + Only Dx			0,882	(0.880 - 0.884)	0,829	(0.826 - 0.832)	0,884	(0.882 - 0.886)
A&S + Only Rx			0,862	(0.859 - 0.864)			0,865	(0.863 - 0.868)
A&S + Dx + Rx			0,889	(0.887 - 0.891)			0,892	(0.890 - 0.894)
A&S + Dx + Rx + cost percentile			0,897	(0.895 - 0.899)	0,869*	(0.866 - 0.871)	0,899	(0.897 - 0.901)
A&S + Dx + Rx + cost percentile + DI			0,897	(0.895 - 0.899)	0,869*	(0.866 - 0.872)	0,899	(0.897 - 0.902)

*The CRG models do not include prescriptions.

Age groups: 14–17; 18–34; 35–44; 45–54; 55–64; 65–69; 70–74; 75–79; 80–84; 85 + .

Cost groups in percentiles: 0–1; 2–10; 11–25; 26–50; 51–75; 76–90; 91–93; 94–95; 96–97; 98 + .

*Dx* diagnoses, *Rx* prescriptions, *DI* Deprivation index.

The inclusion of the deprivation index resulted in a marginal improvement in the coefficients of determination for cost prediction or AUC for identifying patients with high needs.

## Discussion

Our study has revealed that the three case-mix systems used (ACG-PM, CRG and DCG-HCC) show sufficient capability to predict use of health resources and identify people with high needs over the next 12 months in an environment other than that for which they were designed and using other sources of information, namely, a publicly-funded universal coverage system and in the absence of an information system based on the use of claim data.

While age and sex can predict 7% of the variability in cost, models that use clinical variables can predict three times this amount and, when prior costs are added, this value is even higher, reaching 27%. In this case, the observed increase in the coefficients on addition of the deprivation index was very small. Moreover, out-of-the-box models would seem to be a reasonable alternative for use in situations such as organisations that serve small populations or that lack sufficient data for self calibration. DCG-based models provided slightly higher explanatory power and the inability to use prescriptions data in the CRG software was a limitation for making comparisons with the latter. In this study, the use of more complex statistical models than OLS did not provide any benefits.

As regards the ability of models to correctly detect people that require large amounts of health resources, the ACG-PM and DCG-HCC results are relatively similar and are higher than those from CRG. However, regarding the out-of-the-box option, the DCG-HCC models show a better behaviour.

This is the first study to compare three case-mix systems in an entire geographical area, including some social variables. Moreover, unlike other studies conducted in Europe [19,20], it combines patient information from various sources: primary care, hospital admissions and specialist outpatient care.

However, some limitations should be noted. First, our healthcare system has no direct cost-per-patient data; these had to be calculated from the standard prices of provided services and in some cases (principally mental health, rehabilitation, hospital at home and some aspects of day hospital) they could not be obtained.

Moreover, the information sources used are sufficiently complete for obtaining information on diagnoses in hospitalised patients, but not in patients treated in outpatient specialty care or the hospital emergency department. With regard to primary care, as is the case elsewhere, in the Basque Country there is a degree of underreporting of diagnoses and prescriptions in EMRs. Also, diagnoses registered only once were accepted, which may have meant that some annotation or coding errors by doctors was overlooked. However, records of primary care doctors tend to suffer from a lack of sensitivity [21] but not specificity, so we do not consider that this would have exerted a significant effect. In any case, although it is known that the quality of diagnosis [22] and prescription [23] information does influence results, there is no reason to believe that these issues are important enough to significantly affect our results, especially given that a requirement of these adjustment models is their ability to work with imperfect data in the real world [3,4]. The use of population adjustment systems requires the application of administrative databases and other sources of information containing data collected for other purposes, whose limitations are known [24,25] and it is assumed that even in major chronic diseases, for a proportion of patients receiving diagnoses in a year, this information is not repeated in the codes extracted during the following year [3,26].

Finally, a third limitation refers to the social variable used (deprivation index) which, given its ecological character, may underestimate the contribution of individual socioeconomic characteristics.

Our results agree with those found by other authors and support the robustness of these case-mix systems [4,6,27]. They display comparable results despite having been conducted in countries with different types of health systems, groups of people with different characteristics to those of the general population or using different scenarios for analysis, such as truncation of costs above a certain threshold. In our case, no transformation was performed on data to achieve a better fit. No patient was excluded for not being a healthcare user and neither were those who, due to death, did not complete the second year. Although it is commonly accepted that regression-based models are more flexible and predict better than those that are cell-based [4-6], in our case the classification of the population based on diagnoses by CGR categories shows a similar predictive ability to the ACG-PM system, which uses a mixed system of categories and regression.

Beyond the predictive capability of the models, other considerations must be taken into account when assessing a case-mix, such as transparency of their classification methods and their ability to provide useful information from a clinical and not just financial point of view. Although some simple methods, such as the number of conditions or medications [28,29] have been used as a disease burden measure and, in some cases, to predict mortality, health care costs, number of visits or other variables. Nevertheless, models based on these methods would have limited implementation given that they do not provide a comprehensive approach to the actual population’ burden of diseases. Additionally, any estimation based on such models would be seriously affected by the variability in clinical practice patterns among clinical practitioners.

All three systems, employed in our study, categorise the diagnoses into clinically relevant groups (260 ACG-PM, 547 CRG and 1,013 DCG) which in turn can easily be collapsed into a smaller number of groups and show a sufficient degree of granularity. The use of these groups, instead of all the registered ICD-9-CM codes, can decrease the influence of the diagnosis coding practices and also provide a manageable number of groups to identify patients with a given disease or observe the distribution of diseases in population subgroups. The DCG-HCC and ACG-PM systems also include an additional method to identify diseases, based on prescriptions. The records of prescribed medication provide a list of diseases that are being treated, which in our case has been particularly useful for obtaining data from patients who only visit their primary care doctor to get prescriptions, because their condition is stable or because their disease is receiving care in outpatient specialised care facilities. Moreover, the coding of drugs is carried out automatically, which minimises errors and prevents manipulation by clinicians.

In contrast to USA [30] or other European countries [31], in a national health system such as the Basque Country, where the component organisations provide healthcare to all the residents in a geographical area and the competition between them is very limited, the hazard of perverse patient selection practices is impossible. However, risk stratification systems have other applications, such as identification of patients eligible for case-disease and case-management programs. These interventions are designed to improve the quality and efficiency of care, especially those who are at risk of having high future care needs. In any case, identification of the latter group will be of little interest, unless within it those patients can be differentiated whose deterioration can be avoided or mitigated by proactive actions by those that are in unmanageable situations. While previous cost displays high explanatory power, it is strongly influenced by factors other than healthcare needs [32,33] and, except when used as a complement to clinical variables, it does not enable the identification of target populations.

An imperative for the implementation of health programmes is that the entire population benefit. Although case-mix systems use multiple clinical data, other variables such as social variables also influence people’s health and care outcomes. For this reason, a system that considers future care needs based on information from prior use may be unfair if there are social groups whose access to health services is less than their actual need [34]. Consequently, doubts could be raised about ethical issues surrounding the implementation of population stratification, based on the possibility of engendering subtle discrimination against patients in a more severe state of deprivation. In our study, inclusion of the deprivation index did not produce a significant difference in the models’ ability, which is in line with another study carried out in Canada using administrative and survey data to predict the number of doctor visits [35]. In any case, maintaining the social variables is justified as a means of ensuring greater equity in patient selection [36].

This study analysed the predictive ability of models based primarily on diagnoses and prescriptions in the total population over 14 years of age in a geographical region. However, in order to implement management programmes for specific diseases, it would be appropriate to also know the capability of these models when applied to subgroups of patients diagnosed with these diseases. Furthermore, from the implementation of Health Information Technologies (HIT), new data can be obtained that until now were not accessible [37], such as data on following clinical practice guidelines, patient treatment adherence, life habits and risk factors, the incorporation of which will allow a better description of the health of the population and could improve the ability of predictive models.

## Conclusion

Our study has shown that case-mix systems developed in the U.S. can be used in a publicly financed healthcare system with universal health insurance such as that of the Basque Country, to predict consumption of health resources. Also, the additional use of information from diagnoses and prescriptions mitigates some of the limitations attributable to the information systems in use and produces better results. The use of these systems allows the identification of people at risk of requiring high health resource consumption in the future and whose situation is potentially preventable through the implementation of proactive interventions.

## Abbreviations

ACG-PM: Adjusted Clinical Groups – predictive modelling; ATC: Anatomical Therapeutic Chemical system.; AUC: Area under curve; CRG: Clinical Risk Groups; DCG-HCC: Diagnostic Cost Groups/Hierarchical Condition Categories; HIT: Health Information Technologies; ICD-9-CM: International Classification of Diseases, 9th Revision, Clinical Modification; MAPE: Mean Absolute Prediction Error; OLS: Ordinary Least Squares; ROC: Receiver operating characteristic.

## Competing interests

The authors declare that they have no competing interests.

## Authors’ contributions

All authors participated in the design of the study. JFO performed the validation of databases. MM was responsible for statistical analyses. JFO wrote the draft of manuscript. All authors participate in interpretation of data, critically reviewed and gave final approval to the manuscript.

## Pre-publication history

The pre-publication history for this paper can be accessed here:

http://www.biomedcentral.com/1472-6963/13/269/prepub

## Supplementary Material

Additional file 1 Classification systems for patients analysed in the study. Click here for file

Additional file 2 Mean values per patient of admissions, specialised and primary care visits, by age groups. Click here for file
