# Supplementary material for: Predictive risk modelling in the Spanish population: a cross-sectional study
Source: BMC Health Serv Res. 2013 Jul 9;13:269. doi: 10.1186/1472-6963-13-269 (PMC3750562; doi:10.1186/1472-6963-13-269)
Supplement: Additional file 1 — Classification systems for patients analysed in the study. [file 1472-6963-13-269-S1.doc]

Classification systems for patients analysed in the study.

*ACG-PM[[1]](#footnote-2)*

This is a system developed by researchers at Johns Hopkins University that uses information from diagnoses, prescriptions, some relevant procedures and healthcare costs.

The ACGs are 94 self-exclusive categories in which each person is classified based on their age, sex and the combination of diagnoses that were assigned over a 12-month period. Diagnoses are also classified in three other ways: in 264 Expanded Diagnosis Clusters (EDCs) based on the clinical characteristics of the health problem; HOSDOM, identifying pathologies with high risk of hospitalisation in the following year; and a marker for medical frailty.

Furthermore, drugs are classified into 69 Rx-defined Morbidity Groups (Rx-MGs), depending on the pathology that can be identified from them. It is considered that a patient has received a large number of generic drugs, when the annual total is greater than 12.

Not all these groups are included as variables in the predictive models, but 180 are included for diagnosis (34 categories for ACGs, 101 EDCs, 4 categories for HOSDOM number and frailty marker) and 65 for prescriptions (64 Rx-MGs, high number of generic drugs). [[2]](#footnote-3)

The system generates predictions based on the case-mix calibration performed by the authors (out-of-the-box values), in two models, one for under-65s and another for 65s and over.

*Verisk Health’s DxCG Classification system[[3]](#footnote-4)*

It was designed by researchers at Boston University and contains a set of different predictive models, based on the explanatory variables used (age and sex, diagnoses, prescriptions, cost or combinations of the above), populations (commercial, Medicaid or Medicare) and the response variables that are being predicted (total cost, hospitalisation, pharmacy cost).

Diagnosis classifications are established based on clinical homogeneity: the ICD-9-CM codes are categorised into 1,013 Dx-groups, which in turn may be collapsed into 394 Condition Categories (CC), then into 117 Related Condition Categories (RCC) and these into 31 Aggregated Condition Categories (ACC). Although all these groups can be used to describe the morbidity of patient populations, it is the CCs that are used in predictive models.

Similarly, prescribed drugs reveal patients’ health problems through their classification into 203 RxGroups (which are the categories used in predictive models), which in turn may be divided into 18 Aggregated Rx Groups.

In predictive models, in order to reduce the excessive proliferation of codes per patient and the variations caused by the coding habits of individual providers, dx and prescriptions groups are subjected to a hierarchisation process, on the basis of which only those categories that capture the most relevant manifestation of each health problem of a patient are chosen.

For the present study, together with our own calibration models, out-of-the-box models developed for commercial populations were used, based on diagnoses (ID#26), prescriptions (ID#69) or both (ID#71).

*Clinical Risk Groups (CRG)[[4]](#footnote-5)*

This system, developed by 3M, uses diagnoses and procedures. In this study it was not possible to add information on prescriptions, since the Basque Country organisation uses the WHO ATC system, which is rejected by the CRG v1.6 software.

The process of grouping diseases is done in several phases: first the ICD-9-CM diagnosis codes are classified into 537 EDCs (Episode Disease Categories) and the procedures into 640 EPCs (Episode Procedure Categories); the combination of EDCs and EPCs and their temporal sequence can lead to some of them being generated or eliminated. The most relevant diseases are then selected and, depending on their characteristics and the combination of other diagnoses, a severity level is established. Finally, each patient is classified into a single CRG from the combination of diagnostic groups. The CRGs are 1,076 self-exclusive categories, which can be regrouped into thirds, to achieve the desired level of granularity.

For out-of-the-box estimates, weights from a Canadian population were used, provided by the software owner company.

1. Johns Hopkins University, School of Public Health: The Johns Hopkins University ACG Case-Mix System. Internet address: http://www.acg.jhsph.org/index.php?option=com_content&view=article&id=46&Itemid=61. Accessed August 21, 2012 [↑](#footnote-ref-2)
2. According to ACG System Technical Reference Guide (Health Services Research & Development Center at Johns Hopkins University, Bloomberg School of Public Health*: The Johns Hopkins ACG® System Reference Guide. Version 9.0*. Baltimore, MD; 2009) the variables included in calibrated models were:

   Individual ACGs: 4220; 4330; 4420; 4430; 4510; 4520; 4610; 4620; 4730; 4830; 4910; 4920; 4930; 4940; 5010; 5020; 5030; 5040; 5050; 5060; 5070; 5320; 5321; 5322; 5330; 5331; 5332; 5340; 5341; 5342

   ACGs included as RUBs : ACG RUB Level (ACG: 0100; 0200; 0300; 1100; 1200; 1600; 5100; 5110; 5200; 9900) ; ACG RUB Level 2 (ACG: 0400; 0500; 0600; 0900; 1000; 1300; 1800; 1900; 2000; 2100; 2200; 2300; 2400; 2500; 2800; 2900; 3000; 3100; 3400; 3900; 4000; 1711; 1721; 1731; 1741); ACG RUB Level 3 (ACG 0700; 0800; 1400; 1500; 2600; 2700; 3200; 3300; 3500; 3600; 3700; 3800; 4100; 4210; 4310; 4320; 4410; 4710; 4720; 4810; 4820; 5310; 1751; 1761; 1771)

   Pregnancy Without Delivery: ACGs (1712, 1722, 1732, 1742, 1752, 1762 and 1772 collapsed into 1 Boolean marker).

   EDCs : ADM03; ALL04; ALL05; ALL06; CAR03; CAR04; CAR05; CAR06; CAR07; CAR09; CAR10; CAR12; CAR13; CAR14; CAR15; END02; END06; END07; END08; END09; EYE03; EYE13; EYE15; GAS02; GAS04; GAS05; GAS06; GAS10; GAS11; GAS12; GSI08; GSU11; GSU13; GSU14; GTC01; GUR04; HEM01; HEM03; HEM05; HEM06; HEM07; INF04; INF08; MAL02; MAL03; MAL04; MAL06; MAL07; MAL08; MAL09; MAL10; MAL11; MAL12; MAL13; MAL14; MAL15; MAL16; MAL18; MUS03; MUS10; MUS14; MUS16; NUR03; NUR05; NUR06; NUR07; NUR08; NUR09; NUR11; NUR12; NUR15; NUR16; NUR17; NUR18; NUR19; NUT02; PSY01; PSY02; PSY03; PSY05; PSY07; PSY08; PSY09; PSY12; REC01; REC03; REC04; REN01; REN02; REN03; REN04; RES02; RES03; RES04; RES08; RES09; RES10; RHU01; RHU05; TOX02; TOX04

   Rx-MGs: ALLx010; ALLx030; ALLx040; ALLx050; CARx010; CARx020; CARx030; CARx040; CARx050; EARx010; ENDx010; ENDx020; ENDx030; ENDx040; ENDx050; ENDx060; ENDx070; EYEx010; EYEx020; EYEx030; FREx010; FREx020; FREx030; GASx010; GASx020; GASx030; GASx040; GASx050; GASx060; GSIx010; GSIx020; GSIx030; GSIx040; GURx010; GURx020; HEMx010; INFx010; INFx020; INFx030; INFx040; INFx050; MALx010; MUSx010; MUSx020; NURx010; NURx020; NURx030; NURx040; NURx050; PSYx010; PSYx020; PSYx030; PSYx040; PSYx050; PSYx060; RESx010; RESx020; RESx030; RESx040; SKNx010; SKNx020; SKNx030; TOXx010; ZZZx000

   Special Population Marker: Boolean indicators for 0 HOSDOM; 1 HOSDOM ; 2 HOSDOM; 3+ HOSDOM3; Frailty ; indicator for 13 or more Generic Drugs:

   Demographic Markers: Female Boolean indicator; Age groups: 0-4; 5-11; 12-17; 18-34; 35-44; 45-54; 55-69; 70-74; 75-79; 80-84; 85+

   Prior Cost Marker: Total Expense: Non-users ; 1-10th Percentile; 11-25th Percentile; 26-50th Percentile ; 51-75th Percentile; 76-91st Percentile; 91-93rd Percentile; 94-95th Percentile; 96-97th Percentile; 98-99th Percentile;

   Pharmacy Expense, Non-users; ; 1-10th Percentile; 11-25th Percentile; 26-50th Percentile; 51-75th Percentile; 76-91st Percentile; 91-93rd Percentile; 94-95th Percentile; 96-97th Percentile; 98-99th Percentile [↑](#footnote-ref-3)
3. Verisk Health’s DxCG DCG_Methodology . Internet address:

   http://www.dxcg.com/resources/library. Accessed August 21, 2012 [↑](#footnote-ref-4)
4. 3M™ Clinical Risk Grouping Software. Internet address: http://solutions.3m.com/wps/portal/3M/en_US/3M_Health_Information_Systems/HIS/Products/CRG/. Accessed August 21, 2012 [↑](#footnote-ref-5)
