# Supplementary material for: Predictive risk modelling in the Spanish population: a cross-sectional study
Source: BMC Health Serv Res. 2013 Jul 9;13:269. doi: 10.1186/1472-6963-13-269 (PMC3750562; doi:10.1186/1472-6963-13-269)
Supplement: Additional file 2 — Mean values per patient of admissions, specialised and primary care visits, by age groups. [file 1472-6963-13-269-S2.doc]

Mean values per patient of admissions, specialised and primary care visits, by age groups.

|  |  | Year-1 | | Year-2 | |
| --- | --- | --- | --- | --- | --- |
|  | Age groups | Mean* | % Patients with one or more admissions or contacts* | Mean | % Patients with one or more admissions or contacts |
|  |  |  |  |  |  |
| No. of hospitalisations | | 0.10 | 7.16% | 0.11 | 7.77% |
|  |  |  |  |  |  |
|  | 14-24 | 0.03 | 2.78% | 0.03 | 2.85% |
|  | 25-34 | 0.06 | 5.61% | 0.07 | 5.83% |
|  | 35-44 | 0.06 | 5.19% | 0.06 | 4.85% |
|  | 45-54 | 0.06 | 4.73% | 0.07 | 5.08% |
|  | 55-64 | 0.10 | 7.17% | 0.11 | 7.88% |
|  | 65-74 | 0.17 | 11.52% | 0.19 | 13.02% |
|  | 75-84 | 0.25 | 17.01% | 0.30 | 19.50% |
|  | 85+ | 0.23 | 16.10% | 0.29 | 19.78% |
|  |  |  |  |  |  |
| Hospitalisation days | | 0.53 |  | 0.63 |  |
|  |  |  |  |  |  |
|  | 14-24 | 0.14 |  | 0.13 |  |
|  | 25-34 | 0.24 |  | 0.23 |  |
|  | 35-44 | 0.26 |  | 0.26 |  |
|  | 45-54 | 0.32 |  | 0.36 |  |
|  | 55-64 | 0.56 |  | 0.67 |  |
|  | 65-74 | 1.03 |  | 1.25 |  |
|  | 75-84 | 1.68 |  | 2.11 |  |
|  | 85+ | 1.68 |  | 2.26 |  |
|  |  |  |  |  |  |
| Emergency visits | | 0.31 | 19.62% | 0.32 | 19.69% |
|  |  |  |  |  |  |
|  | 14-24 | 0.35 | 22.20% | 0.34 | 21.96% |
|  | 25-34 | 0.35 | 21.70% | 0.35 | 21.22% |
|  | 35-44 | 0.28 | 17.88% | 0.26 | 16.94% |
|  | 45-54 | 0.23 | 15.20% | 0.22 | 14.99% |
|  | 55-64 | 0.25 | 16.49% | 0.25 | 16.66% |
|  | 65-74 | 0.33 | 20.13% | 0.35 | 21.16% |
|  | 75-84 | 0.47 | 26.67% | 0.52 | 28.87% |
|  | 85+ | 0.50 | 27.80% | 0.58 | 30.89% |
|  |  |  |  |  |  |
|  |  |  |  |  |  |
| Specialty outpatient visits | | 1.70 | 43.53% | 1.76 | 44.28% |
|  |  |  |  |  |  |
|  | 14-24 | 0.83 | 31.41% | 0.84 | 31.34% |
|  | 25-34 | 1.02 | 32.43% | 1.08 | 33.48% |
|  | 35-44 | 1.19 | 35.22% | 1.23 | 35.89% |
|  | 45-54 | 1.57 | 42.12% | 1.66 | 43.41% |
|  | 55-64 | 2.20 | 51.56% | 2.30 | 52.98% |
|  | 65-74 | 2.98 | 62.55% | 3.12 | 63.69% |
|  | 75-84 | 3.29 | 66.90% | 3.30 | 66.77% |
|  | 85+ | 1.74 | 46.29% | 1.57 | 43.41% |
|  |  |  |  |  |  |
| Nurse visits | | 1.69 | 32.20% | 2.09 | 38.33% |
|  |  |  |  |  |  |
|  | 14-24 | 0.41 | 17.34% | 0.53 | 22.02% |
|  | 25-34 | 0.56 | 18.18% | 0.72 | 22.77% |
|  | 35-44 | 0.61 | 19.03% | 0.75 | 23.48% |
|  | 45-54 | 0.96 | 25.34% | 1.21 | 31.64% |
|  | 55-64 | 2.09 | 41.84% | 2.65 | 51.25% |
|  | 65-74 | 3.83 | 60.77% | 4.72 | 70.06% |
|  | 75-84 | 5.26 | 65.47% | 6.40 | 72.56% |
|  | 85+ | 5.10 | 56.69% | 5.81 | 58.04% |
|  |  |  |  |  |  |
| Primary health care doctor visits | | 4.47 | 70.32% | 4.29 | 70.26% |
|  |  |  |  |  |  |
|  | 14-24 | 2.58 | 65.09% | 2.64 | 66.38% |
|  | 25-34 | 3.00 | 63.74% | 2.92 | 63.70% |
|  | 35-44 | 3.18 | 63.35% | 3.09 | 63.07% |
|  | 45-54 | 3.80 | 66.71% | 3.79 | 67.47% |
|  | 55-64 | 5.34 | 76.97% | 5.16 | 77.71% |
|  | 65-74 | 7.03 | 84.52% | 6.62 | 84.39% |
|  | 75-84 | 8.63 | 85.48% | 7.96 | 83.48% |
|  | 85+ | 7.54 | 71.57% | 6.50 | 65.27% |
|  |  |  |  |  |  |
| Healthcare cost | | €899.68 |  | €1117.25 |  |
|  |  |  |  |  |  |
|  | 14-24 | 330.28 |  | 366.63 |  |
|  | 25-34 | 432.10 |  | 482.82 |  |
|  | 35-44 | 483.83 |  | 537.82 |  |
|  | 45-54 | 646.55 |  | 792.35 |  |
|  | 55-64 | 1078.36 |  | 1361.85 |  |
|  | 65-74 | 1728.79 |  | 2229.60 |  |
|  | 75-84 | 2365.06 |  | 3076.07 |  |
|  | 85+ | 2067.14 |  | 2734.73 |  |

*People who died in year 1 are excluded, since it makes no sense to predict resource consumption for year 2. For this reason, there appears to be a greater number of admissions and hospital stays in the first year. Between 2008 and 2009 the healthcare cost rose by 9.8%
